# Perceptual Distortions in Pitch and Time Reveal Active Prediction and Support for an Auditory Pitch-Motion Hypothesis

**DOI:** 10.1371/journal.pone.0070646

**Published:** 2013-08-06

**Authors:** Molly J. Henry, J. Devin McAuley

**Affiliations:** 1 Michigan State University, East Lansing, Michigan, United States of America; 2 Bowling Green State University, Bowling Green, Ohio, United States of America; CEA.DSV.I2BM.NeuroSpin, France

## Abstract

A number of accounts of human auditory perception assume that listeners use prior stimulus context to generate predictions about future stimulation. Here, we tested an auditory pitch-motion hypothesis that was developed from this perspective. Listeners judged either the time change (i.e., duration) or pitch change of a comparison frequency glide relative to a standard (referent) glide. Under a constant-velocity assumption, listeners were hypothesized to use the pitch velocity (Δf/Δt) of the standard glide to generate predictions about the pitch velocity of the comparison glide, leading to perceptual distortions along the to-be-judged dimension when the velocities of the two glides differed. These predictions were borne out in the pattern of relative points of subjective equality by a significant three-way interaction between the velocities of the two glides and task. In general, listeners’ judgments along the task-relevant dimension (pitch or time) were affected by expectations generated by the constant-velocity standard, but in an opposite manner for the two stimulus dimensions. When the comparison glide velocity was faster than the standard, listeners overestimated time change, but underestimated pitch change, whereas when the comparison glide velocity was slower than the standard, listeners underestimated time change, but overestimated pitch change. Perceptual distortions were least evident when the velocities of the standard and comparison glides were matched. Fits of an imputed velocity model further revealed increasingly larger distortions at faster velocities. The present findings provide support for the auditory pitch-motion hypothesis and add to a larger body of work revealing a role for active prediction in human auditory perception.

## Introduction

An emerging view of human perceptual and cognitive processes assumes that individuals actively generate predictions about both the ‘what’ and ‘when’ of impending stimulation [1]–[5]. When predictions are satisfied, perception (e.g., detection of a stimulus or discrimination between two stimuli) is facilitated [6]–[8]. Moreover, neural responses (e.g., event-related potentials in EEG/MEG or BOLD response in fMRI) are attenuated when sensory events are predicted relative to when sensory events are unexpected [9]. Attenuated neural responses to predicted events are hypothesized to reflect more efficient processing relative to sensory events that are inconsistent with internally-generated predictions.

Naturally-occurring sounds are often characterized by temporal and spectral regularities that allow listeners, in principle, to predict how the sound will unfold in time. In the current work, we tested an *auditory pitch-motion hypothesis* [4], [10]–[13], which proposes that listeners are sensitive to the pitch motion of a stimulus [14] and moreover use an assumption of constant pitch velocity (Δf/Δt) to generate expectations about the future time course of the stimulus. Critically, when expectations generated from the velocity trajectory are violated, perceptual distortions are predicted to result, which reflect the nature of the listeners’ expectations. In what follows, we review the assumptions of the auditory pitch-motion hypothesis and describe a mathematical model that quantifies the contribution of velocity-based expectations to perceptual distortions.

### The Auditory Pitch-Motion Hypothesis

Here, we use the term *auditory pitch-motion* to refer to motion of an auditory stimulus in frequency space. We note that previous studies have used the term *auditory motion*. However, we prefer to refer to auditory pitch-motion in order to avoid potential confusions with motion of an auditory stimulus in physical space. Auditory pitch motion conveys important information to listeners in both music and speech contexts [4], [12], [15]–[18]. For example, in music, the notes of a melody change in frequency in systematic ways to convey information about tonality [17] and contribute to perceived accent structure [19]. In the speech domain, patterns of rises and falls in pitch over time (an element of speech prosody) help determine word boundaries, distinguish between questions and statements, disambiguate semantic content, place emphasis, and convey information about the emotional state of the speaker [15], [16], [18]. Of interest here is the general hypothesis that auditory pitch motion provides listeners a framework from which to predict the future time-course of a stimulus, that is, ‘what’ will happen ‘when’.

The auditory pitch-motion hypothesis proposes that listeners are sensitive to the perceived pitch velocity of an auditory stimulus [14], where pitch velocity, *V*, is approximated by Δf/Δt. Throughout this article, we will use Δf to refer to the change in frequency in units of Hertz (Hz) between two time points, namely the onset and offset of a stimulus, while Δt refers to the amount of time that has elapsed between the same two time points and as such is equivalent to the duration of the stimulus.

Two critical tenets of the auditory pitch-motion hypothesis are that (1) listeners assume that auditory stimuli will tend to maintain a relatively constant pitch velocity and (2) listeners use this information to generate expectations about the future timing and pitch characteristics of an unfolding stimulus. As such, an unexpected increase in the pitch distance covered per unit time is predicted to lead to the perceived expansion of the corresponding time interval in order to equalize perceived velocity across the stimulus. Conversely, an unexpected decrease in the duration of a stimulus interval is predicted to lead to a subjective shrinking of perceived pitch distance of the same interval, again so that the stimulus conforms to the expectation of constant velocity. Moreover, an unexpected decrease in the frequency change across an interval should lead to a subjective shrinking of perceived duration, while an unexpected increase in frequency change is predicted to lead to a subjective expansion of the corresponding duration.

**Figure 1:**
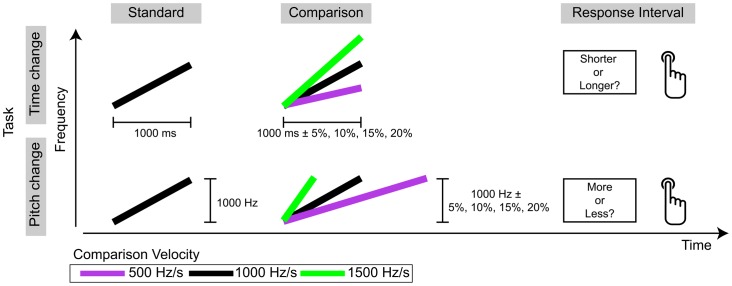
Schematic of stimuli and task for the current experiment. Here, only the 1000-Hz velocity condition is illustrated. The time-change (i.e., duration-judgment) task is shown on the top line, and the pitch-change task is shown on the bottom line. On each trial, a constant-velocity standard glide was presented. Then a variable-velocity comparison stimulus was presented; the comparison velocity took on one of three values. Comparison levels were varied parametrically on the to-be-judged dimension; thus values of the ignored dimension were adjusted in order to maintain the prescribed velocity. For the time-change task, listeners indicated whether the comparison was shorter or longer than the standard. For the pitch-change task, listeners indicated whether the comparison changed more or less in pitch than the standard.

In this study, we tested these two tenets of the auditory pitch-motion hypothesis by having listeners judge either the perceived time change (i.e., duration) or perceived pitch change of a comparison stimulus relative to the perceived duration or pitch change of a referent (standard) stimulus. Both the standard and comparison stimuli were frequency glides that changed linearly in frequency over their temporal extent at a constant velocity (Fig. 1). For each listener, the velocity of the standard glide was fixed at one of three values (500 Hz/s, 1000 Hz/s, or 1500 Hz/s) to provide a constant-velocity referent. Based on the auditory pitch-motion hypothesis, listeners’ were assumed to use the velocity of the standard glide to generate predictions about the amount of time change or frequency change that would be expected to occur between the onset and offset of the comparison glide. To either violate or reinforce the constant velocity assumption, the comparison velocity varied from trial to trial and took on values of 500 Hz/s, 1000 Hz/s, and 1500 Hz/s. This meant that in some cases, the standard and comparison velocities matched, and in other cases, they did not.

### Predictions

Predictions for both tasks were generated by formalizing the auditory pitch-motion hypothesis in terms of an *imputed velocity model* [10], [11], [20]. In this model, perceived duration and pitch change are represented as a weighted combination of actual and expected values along the to-be-judged dimensions. Expected values for both tasks are derived from the assumption that participants use the constant (referent) velocity, *V*, established by the standard glide when asked to judge stimulus attributes of the comparison glide. For the time change task, the perceived duration of the comparison glide, *τ*, is given by:
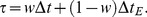
(1)


Here, the actual duration of the comparison glide is represented by Δ*t*, and the expected duration of the comparison glide is represented by Δ*t_E_* where Δ*t_E_* = Δ*f/V*. For the pitch-change task, the perceived pitch change, *φ*, of the comparison glide is given by:
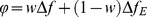
(2)


Here, the actual frequency change of the comparison glide is represented by Δ*f* and the expected frequency change of the comparison glide is represented by Δ*f_E_,* where Δ*f_E_ = *Δ*tV.* Equations 1 and 2 [20] reflect the idea that perceived time change or pitch change can be estimated from a weighted combination of actual and expected values, where for both tasks, the expected values along the to-be-judged dimension are derived from the standard equation for velocity, *V = *Δ*f/*Δ*t;* the form differs simply because the computed expected value (Δ*f_E_* or Δ*t_E_*) is in the numerator for the pitch-change task, but in the denominator for the time-change task. Nonetheless, for both tasks, perceptions along the to-be-judged dimension are distorted in the direction of the constant velocity established by the standard (referent) glide. The weight value, *w* ∈ [0,1], represents a free parameter that can be used to estimate the magnitude of these perceptual distortions due to imputed velocity, with smaller values of *w* corresponding to larger perceptual distortions.

The auditory pitch-motion hypothesis and associated imputed pitch velocity model make the following behavioral predictions for the time-change and pitch-change tasks. In general, the predictions for both tasks are based on the view that perceived duration and pitch change of the comparison glide will reflect both the actual values (Δ*t* or Δ*f* depending on the task-relevant dimension) and the expected values derived from the velocity of the standard glide (Δ*t_E_* = Δ*f/V or* Δ*f_E_ = *Δ*tV*). Note that for both tasks, when the velocities of the standard and comparison glides are the same, the actual and expected values of the comparison glide along the task-relevant dimension are equal (Δ*t = *Δ*t_E_* or Δ*f_E_ = *Δ*f*) and in this case no systematic perceptual distortions are predicted. However, systematic perceptual distortions are predicted for both tasks with the velocities of the two glides differ.

For the time-change task, when the velocity of the comparison glide is faster than the standard, the comparison changes more in frequency per unit time than expected given the velocity of the standard, and thus the expected duration of the comparison is longer than its actual duration (Δ*t_E_*>Δ*t*). In this case, the perceived duration of the comparison is predicted to be overestimated. On the other hand, when the velocity of the comparison glide is slower than the standard, the comparison changes less in frequency per unit time than expected given the velocity of the standard, and thus the expected comparison duration is shorter than actual duration (Δ*t_E_*<Δ*t*). In this case, the perceived duration of the comparison is predicted to be underestimated.

Conversely, for the pitch-change task, when the velocity of the comparison glide is faster than the standard, the duration of the comparison is shorter than would be expected given the velocity the standard. Thus the expected value for pitch change is less than the actual value (Δ*f_E_*<Δ*f*), and perceived pitch change is predicted to be underestimated. Conversely, when the comparison velocity is slower than the standard, comparison time change is longer than standard time change. Thus the expected value for pitch change is greater than the actual value (Δ*f_E_*>Δ*f*), and comparison pitch change is expected to be systematically overestimated.

In sum, the auditory-pitch motion hypothesis and associated imputed velocity model make opposite patterns of predictions for the time-change and pitch-change tasks. When the velocities of the comparison and standard glides match, then no systematic distortions in perceived duration or pitch change are predicted. However when the velocity of the comparison glide is faster than the standard, perceived duration and pitch change are predicted to be over- and underestimated, respectively. In contrast, when the velocity of the comparison glide is slower than the standard, perceived duration and pitch change are predicted to be under- and overestimated, respectively. In the context of the design of the present study, in which we separately manipulated the velocities of the standard and comparison glides, the predictions of the auditory pitch-motion hypothesis translate into the expectation of a three-way interaction between standard velocity, comparison velocity, and task (time-change vs. pitch change).

## Materials and Methods

### Ethics Statement

The Institutional Review Board of Michigan State University approved all procedures, which were in accordance with the Declaration of Helsinki. Written informed consent was obtained from all participants prior to the experiment.

### Design

The design of the experiment was a 2 (Task: time-change, pitch-change)×3 (Standard Velocity: 500 Hz/s, 1000 Hz/s, 1500 Hz/s)×3 (Comparison Velocity: 500 Hz/s, 1000 Hz/s, 1500 Hz/s)×8 (Comparison Level: −20%, −15%, −10%, −5%, +5%, +10%, +15%, +20%) mixed factorial. Task and Standard Velocity were between-participants factors, while Comparison Velocity and Comparison Level were within-participant factors. Participants judged either the time change (i.e., duration) or pitch change of a variable-velocity comparison stimulus relative to a constant-velocity standard stimulus.

### Participants

One hundred and eight individuals (80 female; ages 18–35 years) from Michigan State University participated in return for course credit in an introductory psychology class. Participants self-reported normal hearing and varied in number of years of formal music training (0–15 years, *M = *3.8, *SD = *3.7). Participants were randomly assigned to one of the three standard velocity conditions (500 Hz/s, n = 36; 1000 Hz/s, n = 40; 1500 Hz/s, n = 32) and completed either the time-change task (n = 56) or the pitch-change task (n = 52).

### Stimuli

Stimuli were pure-tone linear frequency glides, ramped linearly over the first and last 5 ms to eliminate acoustic artifacts. Standard glides ascended in pitch at one of three velocities (500 Hz/s, 1000 Hz/s, 1500 Hz/s); for each fixed standard velocity condition, comparison glides also ascended in pitch at one of three velocities (500 Hz/s, 1000 Hz/s, 1500 Hz/s). For the time-change task, the standard glide was always 1000 ms in duration and the comparison glide took on one of 8 values with equal probability (1000 ms ±5%, 10%, 15%, 20%). For the pitch-change task, the change in frequency for the standard glide was always 1000 Hz and the comparison glide took on one of 8 values with equal probability (1000 Hz ±5%, 10%, 15%, 20%). Thus, for all combinations of standard and comparison velocities, participants completing each task experienced the same standard and set of comparisons along the task-relevant dimension. In order for that to be the case, the value of the comparison along the task-irrelevant dimension necessarily varied in order to maintain the prescribed velocity. The starting frequencies of the standard and comparison glides were independently randomized from trial to trial, taking on one of three values (476 Hz, 600 Hz, 756 Hz).

### Apparatus

Stimuli were generated using MATLAB software (The Mathworks, Inc.). Stimulus generation and response collection were controlled using E-Prime 2.0.8.73 software (Psychology Software Tools, Inc.) running on Dell Optiplex computers. Auditory stimuli were presented at a comfortable listening level (∼70 dB SPL) over Sennheiser HD 280 Pro headphones. Responses were made using a serial response box; “shorter” vs. “longer” and “more pitch change” vs. “less pitch change” responses were made by pressing left and right buttons, respectively.

### Procedure

On each trial, participants heard a pair of glides and judged the time change or pitch change of the comparison (second) stimulus relative to the standard (first). For the time-change task, participants judged whether the comparison was “shorter” or “longer” than the standard, and for the pitch-change task participants judged whether the comparison changed “more” or “less” in pitch than the standard (Fig. 1). Before completing the experiment proper, participants heard recorded instructions and completed an 18-trial training block with corrective feedback. During training, participants heard only comparison levels of ±20%. Participants then completed two experimental blocks with no feedback; a short break was allowed between blocks. In each block, participants provided three responses to each combination of Comparison Velocity and Comparison Level, for a total of 72 trials. Overall, listeners completed a total of 144 trials; thus 6 observations were obtained for each Comparison Velocity×Comparison Level combination. The experiment lasted approximately 30 minutes.

### Data Analysis

Proportions of “longer” responses (time-change task) and “more pitch change” responses (pitch-change task) were determined for each participant for each of the eight values of Comparison Level for each Comparison Velocity, averaged over the two test blocks. Relative points of subjective equality (PSEs) for each of the resulting psychometric curves were then estimated for each participant using the following procedure, which fits response proportions to a cumulative normal (Gaussian) probability density function [21]. First, response proportions were converted to z-scores. A correction was applied to proportions of 0 or 1 so that they could be z-transformed; 0 and 1 proportions were first converted to 1/2N or 1–(1/2N), respectively, where N = 6 trials per condition. After z-transformation (which results in psychometric curves becoming approximately linear), we estimated the best-fit straight line through the eight data points making up the psychometric curve using a least-squares approach. From the resulting best-fit linear equation, we estimated the relative point of subjective equality (PSE), which corresponds to the value of the comparison stimulus along the judged dimension which yielding 50% “longer” or “more pitch change” responses, respectively. Note that PSE is typically expressed in units corresponding to the physical stimulus dimension being manipulated (i.e., Hz or ms). However, we relativized our stimulus dimensions and expressed them as percentage changes with respect to the value of the standard stimulus so that relative PSEs would be comparable across tasks. The relative PSE measure quantifies the degree to which perceived duration or pitch change was under- or overestimated relative to the point of objective equality; in signal detection terms, relative PSE is measure of perceptual bias, with negative values indicating overestimation, while positive values indicate underestimation.

Proportions of “longer” and “more pitch change” responses were also fit with the imputed velocity model. For each participant, we simultaneously fit three psychometric functions corresponding to the three comparison velocity conditions. That is, separately for each task and standard velocity, the model simultaneously took into account the eight comparison levels and three comparison velocities; thus *w* values were estimated on the basis of 24 data points (6 observations per point). Specifically, predicted response proportions were obtained from the following equation:
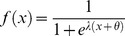
(3)where *x* is a vector of perceived duration or pitch change at all comparison levels and all comparison velocites estimated from equations 1 and 2 [10]. Model fits estimated the best-fitting value of the weight parameter, *w*, by minimizing root mean square error between observed response proportions and response proportions predicted by equation 3. Mean *w* values are reported for the time-change and pitch-change tasks separately for all standard velocity conditions.

## Results

**Figure 2:**
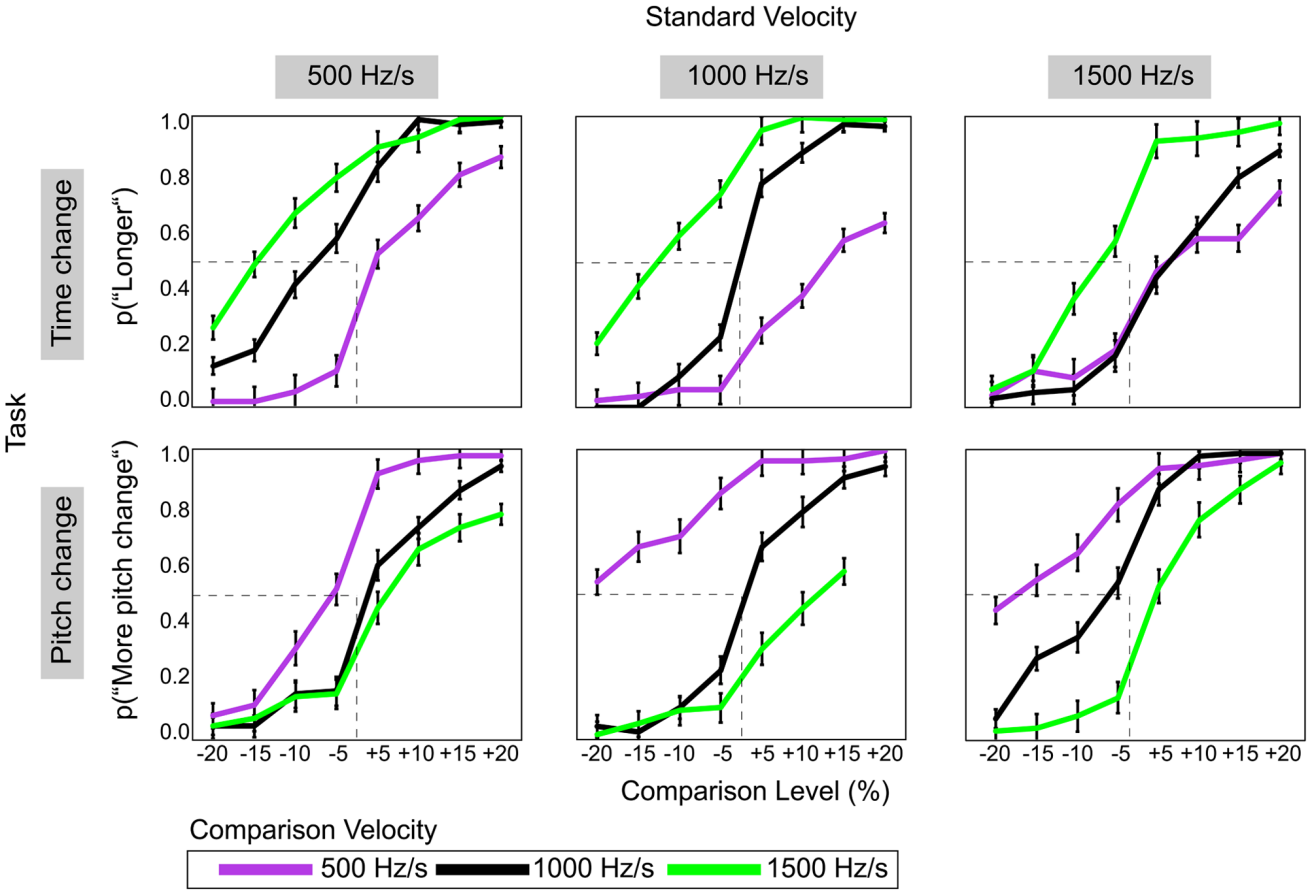
Proportions of “longer” or “more pitch change” responses as a function of Comparison Level for the time-change (top) and pitch-change (bottom) tasks, and shown separately for the three standard velocity conditions. Overall, duration tended to be overestimated when the comparison velocity was faster than the standard velocity, and underestimated when the comparison was slower than the standard. On the other hand, pitch change was underestimated when the comparison was relatively fast and overestimated when the comparison was relatively slow. Notably, results were opposite for the time-change and pitch-change tasks.

**Figure 3:**
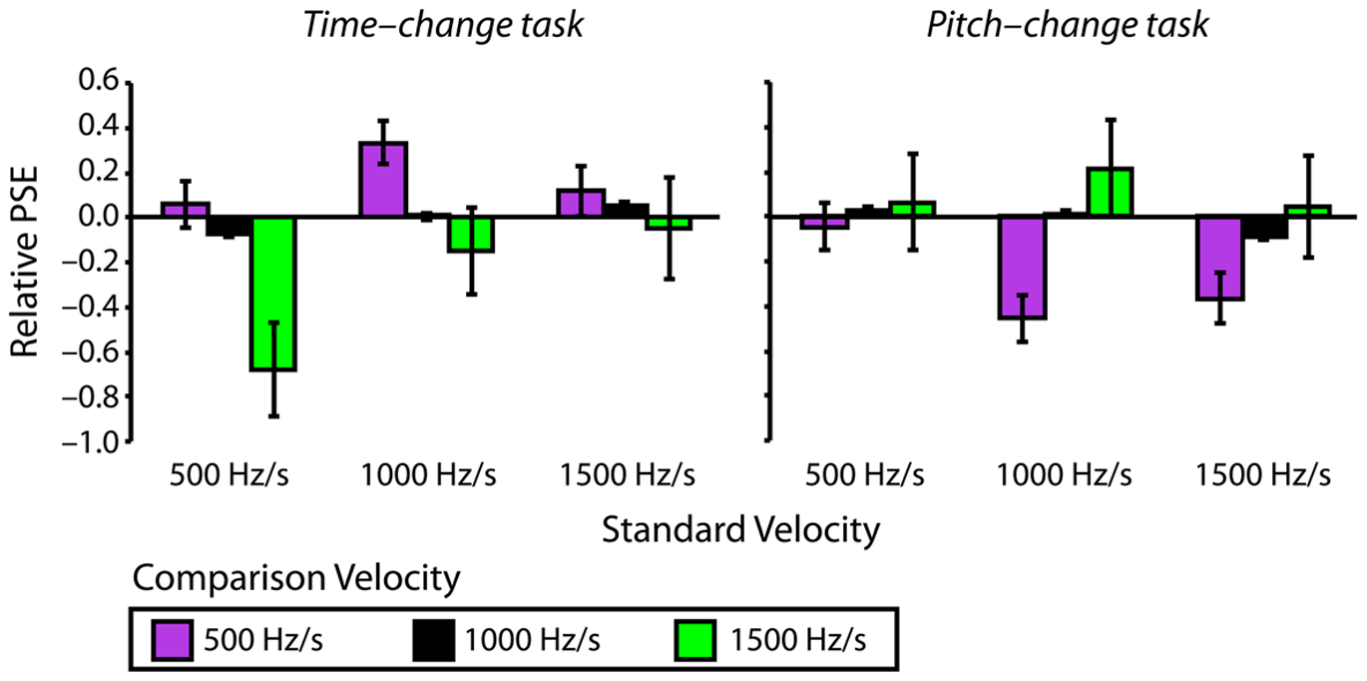
Relative PSEs shown separately for the time-change (left) and pitch-change (right) tasks, separately for each standard and comparison velocity condition; comparison velocities are indicated by different color bars. The plot shows the three-way interaction predicted by the auditory pitch-motion hypothesis.

Figure 2 shows proportions of “longer” responses (time-change task) and “more pitch change” responses (pitch-change task) as a function of relative Comparison Level and the velocity of the comparison glide (500 Hz/s, 1000 Hz/s, 1500 Hz/s) for each Standard Velocity condition (500 Hz/s, 1000 Hz/s, 1500 Hz/s). Corresponding values of relative PSE are reported in Figure 3. For all three standard glide velocities, perceived duration and pitch change were relatively undistorted when the velocities of the standard and comparison glides were equal (i.e., matched). However, when the velocities of the standard and comparison glides differed, there were systematic distortions in perceived duration and pitch change that were in opposite directions, as predicted by the auditory pitch-motion hypothesis. Across all three Standard Velocity conditions, psychometric curves were left-shifted for the time-change task (duration was overestimated) and right-shifted for the pitch-change task (pitch change was underestimated) when the velocity of the comparison glide was faster than the velocity of the standard glide, while the reverse was true when the velocity of the comparison glide was slower than the velocity of the standard glide (Fig. 2).

With respect to relative PSE values, the auditory pitch-motion hypothesis critically predicts a three-way interaction between Task, Standard Velocity, and Comparison Velocity. A Task×Standard Velocity×Comparison Velocity mixed-measures Analysis of Variance (ANOVA) on relative PSEs yielded the predicted three-way interaction, *F*(2.6, 134.1) = 1.02, *MSE = *0.53, *p = *.02, η^2^_p_ = .02. This interaction is indicative of relative PSEs varying systematically based on the combination of standard and comparison velocities, but in an opposite fashion for the time-change and pitch-change tasks. In general, relative PSEs were negative for the time-change task and positive for the pitch-change task when comparison velocity was faster than standard velocity, but the reverse was true when the comparison velocity was slower than the standard velocity. The three-way ANOVA also yielded significant two-way interactions: Task×Comparison Velocity (*F*(2,206) = 14.47, *MSE = *0.53, *p*<.001, η^2^_p_ = .12) and Task×Standard Velocity (*F*(2,103) = 4.19, *MSE = *0.35, *p = *.02, η^2^_p_ = .08). Critically however, these interactions are necessarily qualified by the significant three-way interaction, the form of which was predicted by the auditory pitch-motion hypothesis.

Next, proportions of “longer” and “more pitch change” responses were fit with the imputed velocity model (see Materials and Methods) to yield estimates of *w* that quantified the degree to which perceived duration and pitch change of the comparison glide depended on the velocity of the standard glide. Smaller *w* values indicate larger contributions of expected time change (Δ*t_E_*) or frequency change (Δ*f_E_*) to time-change and pitch-change judgments, respectively. Results are summarized in Table 1. Values of *w* derived from the imputed velocity model were subjected to a 2 (Task)×3 (Standard Velocity) between-subjects ANOVA. Only the main effect of Standard Velocity was significant, *F*(2,108) = 13.01, *MSE = *0.03, *p*<0.001, η^2^_p = _0.19, indicating higher *w* values for the 500 Hz/s standard velocity (*w = *0.88±0.03) than for either the 1000 Hz/s (*w = *0.70±0.04) or 1500 Hz/s standard velocities (*w = *0.77±0.03), *p*s <0.05, which were not different from each other, *p = *0.13, according to Tukey’s HSD post-hoc test.

**Table 1:** Values of w derived from the imputed velocity model, shown separately for the time-change and pitch-change tasks, and for the three Standard Velocity conditions.

		Standard Velocity		
		500 Hz/s	1000 Hz/s	1500 Hz/s
Task	Time-change	0.88 (±0.06)	0.74 (±0.03)	0.79 (±0.03)
	Pitch-change	0.89 (±0.09)	0.69 (±0.04)	0.77 (±0.07)

A significant main effect of Standard Velocity indicated that w values were higher for the 500 Hz/s standard velocity than for either the 1000 Hz/s or the 1500 Hz/s standard velocity condition.

## Discussion

Systematic perceptual distortions were examined for a task where listeners had to judge either the perceived duration or pitch change of glides that varied continuously in frequency at a constant velocity. Of central interest were the predictions of an auditory pitch-motion hypothesis, i.e., that perceptual distortions would be consistent with listeners assuming a constant velocity across the standard–comparison stimulus pair. Consistent with this idea, listeners’ judgments about the task-relevant dimension (time or pitch) were affected by expectations generated based on the constant-velocity standard, but in an opposite manner. In statistical terms, we observed a significant three-way interaction between Task, Standard Velocity, and Comparison Velocity, the form of which was predicted by the auditory pitch-motion hypothesis.

Specifically, perceptual distortions were relatively small when the standard and comparison velocities were matched. For the time-change task, duration was overestimated when comparison velocity was faster than the standard and underestimated when the comparison velocity was slower than the standard. On the other hand, pitch change was underestimated when comparison velocity was faster than the standard and overestimated when the comparison velocity was relatively slow. Thus, as expected, the pattern of distortions was opposite for the time-change and pitch-change tasks.

### Possible Alternative Explanations

We have interpreted the current results as being consistent with the auditory pitch-motion hypothesis. However, in reminder tasks that involve the presentation of a fixed standard on each trial followed by a variable comparison, it has been shown that listeners sometimes ignore the standard altogether, and instead compare the current value of the comparison to a remembered standard [22]. Additionally, when comparison values vary along a continuum, a convergence towards the mean value is often observed such that larger magnitude stimuli tend to be underestimated while smaller magnitude stimuli tend to be overestimated [23]–[25]. Thus, it is important to consider a possible alternative explanation for the current results, which is that listeners ignored the standard stimulus entirely, and the observed perceptual distortions instead resulted from using the average comparison velocity as the constant-velocity referent. Critically, this hypothesis predicts an identical pattern of results across all three Standard Velocity conditions. However, since we observed different patterns of results for the three standard velocities (as indexed by the significant three-way interaction), an explanation based on using an average of comparison velocities as a constant-velocity referent is not viable.

A second potential alternative explanation for the current results concerns the use of the same response button assignment for all listeners in all conditions. That is, “shorter” and “more pitch change” responses were always made using a left-side button, while “longer” and “less pitch change” responses were always made using a right-side button. Previous work has shown stimulus-response compatibility effects for timing [26] and pitch judgments [27]–[29]. That is, responses are faster and more accurate when “faster”/”shorter” responses are made with a left-side button and “slower”/”longer” responses are made with a right-side key. In the pitch domain, an advantage is observed when relatively high and low frequencies are responded to by pressing relatively high and low buttons, respectively, on a vertical button display. These results have been interpreted to reflect a natural mapping of some stimulus dimensions to physical space.

Although a similar effect has not been demonstrated for velocity, it nonetheless seems important to consider whether the results of the present study could be explained by a natural mapping of slow velocities to the left side and fast velocities to the right side. Assuming such a mapping, our results are potentially explainable in part by a bias to press the left button (“shorter”, “more pitch change”) when the comparison velocity was slower than the standard and the right button (“longer”, “less pitch change”) when the comparison velocity was faster than the standard. In order to test this possibility, we replicated the 1000-Hz standard velocity condition from the current experiment with button assignments reversed. The results showed that the stimulus-response mapping does not matter for the tested tasks and thus provide additional support for the auditory pitch-motion hypothesis (see Fig. A and supplemental text in File S1). That is, independent of stimulus-response mapping, duration was overestimated and pitch change underestimated when the comparison velocity was faster than the standard, while duration was underestimated and pitch change overestimated when the comparison velocity was slower than the standard.

### Magnitude of Perceptual Distortions as a Function of Overall Velocity

One prediction that is explicitly made by the auditory pitch-motion hypothesis regards the effect of velocity on the strength of predictions made by listeners about future stimulation, and subsequent perceptual distortions. Specifically, the auditory pitch-motion hypothesis predicts a range of rates within which perceptual distortions should obtain, and in particular, that the magnitude of perceptual distortions should increase with increasing velocity [12]. This hypothesis has previously been supported by experiments requiring listeners to judge either the timing or pitch of a target tone embedded in tone sequences [10], [11]. Both the timing and pitch of the target tone were varied, although only one dimension was task relevant, as in the current study. Similar to the current results, listeners’ perception of target timing and pitch were distorted in the direction consistent with listeners generating expectations about time-pitch location of the target based on assumed constant velocity across the tone sequence. Moreover, perceptual distortions increased in magnitude with increasing stimulus velocity. The current results are concordant with these previous findings, in that here, we observed largest *w* values (and thus smallest perceptual distortions) for the slowest standard velocity condition (500 Hz/s).

### Potential Effects of Glide Direction

In the current study, we examined time-change and pitch-change judgments for glides that ascended in frequency, but not for glides that descended in frequency. We restricted ourselves here to studying ascending glides, because we have directly compared ascending and descending auditory stimuli in a number of previous studies [10], [11], [14], and found very little difference between them. For example, in two studies quantifying the degree to which listeners make use of pitch-velocity information to predict the timing and frequency of a single tone in discrete tone sequences [10], [11], we found that the magnitude of perceptual distortions based on pitch velocity were slightly larger for descending than for ascending tone sequences, but the results were qualitatively the same. We have previously proposed an auditory gravity hypothesis to explain these results. The key assumption of this hypothesis is that descending auditory stimuli are heard as accelerating relative to ascending stimuli; thus, from the perspective of the present study, we might expect perceptual distortions based on pitch velocity to be exaggerated for descending glides, but the overall pattern would be expected to be the same.

An alternative hypothesis for descending glides comes from a study by van Wassenhove and colleagues [30], where the subjective duration of ascending glides was overestimated relative to the duration of descending glides. This is consistent with comparisons of auditory stimuli that increase versus decrease in intensity, and thus sound as if they are approaching or receding, respectively [31]–[34]. Specifically, approaching stimuli are consistently judged as longer than receding stimuli. Thus, it is possible that, in the current study, the duration of ascending glides would be in general overestimated relative to the duration of descending glides. We suspect that behavioral differences between ascending and descending glides in the current study would be unlikely, however, for the following reason. In the current study, duration and pitch-change judgments were relative – that is, they involved judging stimulus attributes of a comparison stimulus relative to a standard that had the same direction, here: ascending). Thus, even if there would be an overall difference between ascending and descending glides in terms of perceived duration, we would expect to see the same three-way interaction between the velocities of the two glides and task. Nonetheless, further studies are needed to conclusively tease apart these two possibilities.

### Predictive Accounts of Auditory Perception

There are several theoretical perspectives that explicitly posit that human perceptual processes are supported by active prediction. For example, predictive coding accounts assume that perceivers make use of statistical regularities in patterns of stimulation in order to generate predictions about upcoming stimuli [1], [2]. On this view, stimulus statistics derived from previous stimulation are specifically used to generate a prior distribution in the Bayesian sense to which unfolding stimuli can be compared. When predictions are violated, an “error signal” results; in terms of dependent measures, the predictive coding account specifically predicts larger neural responses for unexpected (relative to expected) stimulus events. Evidence for this account comes from a number of sources, including for example, mismatch negativity (MMN) paradigms, where a repeated stimulus regimen is occasionally interrupted by a “deviant”, or unexpected, token. EEG and MEG recordings reveal a “mismatch” response to the deviant item occurring after approximately 100–250 ms [9], [35], which is assumed to reflect the error signal [1], [36]. Similarly, BOLD responses observed in fMRI studies are typically larger for unexpected events than for expected events [37], [38].

An alternative, but not necessarily mutually exclusive, account of predictive elements of perceptual processing is dynamic attending theory, which is primarily an account of how expectations can be built up *in time* [4], [7], [39]. In brief, dynamic attending theory posits that attention is an inherently rhythmic process; that is, attention waxes and wanes as time passes. However, the attentional rhythm is easily entrained by external stimulation. Through entrainment, attentional peaks come to be aligned with the expected onset times of important stimulus events; thus, events occurring at expected times are better processed than events occurring at unexpected times. Supporting this idea is a volume of behavioral research showing that the perception of the timing or pitch of an event is better when the event occurs at the expected time based on a previous temporal context [6]–[8], [40], [41]. See ref. [42] for a recent neural framework that explains temporal predictions by appealing to neural oscillations.

The auditory pitch-motion hypothesis is consistent with an extension of dynamic attending theory to a two-dimensional (time-pitch) stimulus space [4], [43]. From this perspective, auditory stimuli varying in pitch over time can be conceptualized as tracing a trajectory through time-pitch space. Deviations from the predicted trajectory then lead to perceptual distortions like those observed here. An important avenue for future research will be to explore how such predictions might be specified neurally, in particular from an oscillatory framework.

### Conclusion

The current study provides a novel test of an auditory pitch-motion hypothesis whereby listeners’ expectations about the velocity of an auditory stimulus were predicted to yield systematic perceptual distortions. Consistent with this prediction, listeners’ judgments about the task-relevant stimulus dimension of a glide (i.e., time or pitch) were influenced by expectations stemming from the constant velocity of a standard (referent) glide. Most striking was support for the prediction that we would observe an opposite pattern of perceptual distortions for the time-change and pitch-change tasks. Time change (i.e., duration) was overestimated when the comparison velocity was faster than the standard and underestimated when the comparison was relatively slow, while pitch change was underestimated when the comparison was fast and overestimated when the comparison velocity was slower than the standard. Overall, the current findings provide further support for the active, predictive nature of human auditory perceptual processing.

## Supporting Information

File S1 Proportions of “longer” or “more pitch change” responses as a function of Comparison Level for the time-change (left) and pitch-change (right) tasks. The data come from a separate experiment in which the 1000 Hz/s standard-velocity condition was repeated, but the mapping of response to buttons was reversed. As we observed in the experiment proper, duration was overestimated when the comparison velocity was faster than the standard velocity, and underestimated when the comparison was slower than the standard. On the other hand, pitch change was underestimated when the comparison was relatively fast and overestimated when the comparison was relatively slow. Notably, results were inconsistent with an explanation based on stimulus-response compatibility effects.
(DOC)
