# Supplementary material for: Perceptual Distortions in Pitch and Time Reveal Active Prediction and Support for an Auditory Pitch-Motion Hypothesis
Source: PLoS One. 2013 Aug 6;8(8):e70646. doi: 10.1371/journal.pone.0070646 (PMC3735601; doi:10.1371/journal.pone.0070646)
Supplement: File S1 — Proportions of “longer” or “more pitch change” responses as a function of Comparison Level for the time-change (left) and pitch-change (right) tasks. The data come from a separate experiment in which the 1000 Hz/s standard-velocity condition was repeated, but the mapping of response to buttons was reversed. As we observed in the experiment proper, duration was overestimated when the comparison velocity was faster than the standard velocity, and underestimated when the comparison was slower than the standard. On the other hand, pitch change was underestimated when the comparison was relatively fast and overestimated when the comparison was relatively slow. Notably, results were inconsistent with an explanation based on stimulus-response compatibility effects. (DOC) [file pone.0070646.s001.doc]

*Supporting Information*

In order to rule out the possibility that the observed perceptual distortions were in part attributable to a bias to press a left-side button (corresponding to “shorter” and “more pitch change” responses) when comparison velocity was relatively fast and a right-side button (corresponding to “longer” and “less pitch change” responses) when comparison velocity was relatively slow, we replicated the 1000 Hz/s standard-velocity condition with the response mappings reversed. For the time-change task, the “shorter” and “longer” responses were made by pushing the right-side and left-side buttons, respectively, and for the pitch-change task, “less pitch change” and “more pitch change” responses were made by pushing the left-side and right-side buttons, respectively.

Figure S1 shows results for this experiment, which involved thirty-nine undergraduates from Michigan State University (n = 20, time-change task; n = 19, pitch-change task). If stimulus-response compatibility effects were responsible for the effects reported in the main manuscript, then the current results should have been critically *opposite* those obtained in original experiment. That is, duration should have been underestimated and pitch-change overestimated for the relatively fast comparison velocity (1500 Hz/s), while duration should have been overestimated and pitch-change underestimated for the relatively slow comparison velocity (500 Hz/s). Instead, the pattern of results was identical to that obtained in Experiment 1. Thus it is considered highly unlikely that stimulus-response compatibility effects could account for our results.


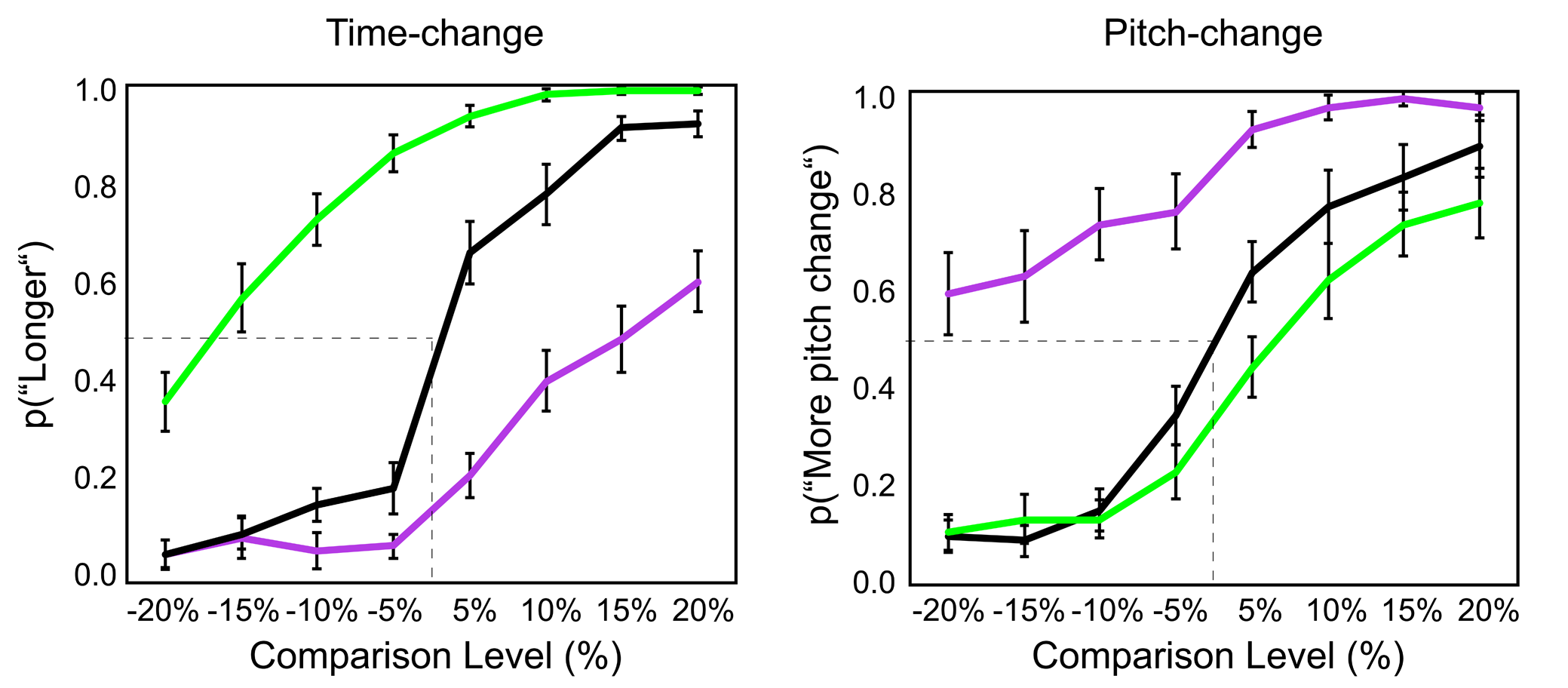


*Figure A.* Proportions of “Longer” or “More pitch change” responses as a function of Comparison Level for the time-change (left) and pitch-change (right) tasks. The data come from a separate experiment in which the 1000 Hz/s standard-velocity condition was repeated, but the mapping of response to buttons was reversed. As we observed in the experiment proper, duration was overestimated when the comparison velocity was faster than the standard velocity, and underestimated when the comparison was slower than the standard. On the other hand, pitch change was underestimated when the comparison was relatively fast and overestimated when the comparison was relatively slow. Notably, results were inconsistent with an explanation based on stimulus-response compatibility effects.
